# RESILIENCE AMONG BLACK FAMILY CAREGIVERS OF PERSONS WITH DEMENTIA

**DOI:** 10.1093/geroni/igad104.0145

**Published:** 2023-12-21

**Authors:** Sheria Robinson-Lane

**Affiliations:** University of Michigan School of Nursing, Ann Arbor, Michigan, United States

## Abstract

Black family caregivers of persons with dementia often experience elevated levels of stress related to lower incomes, chronic health conditions, and care recipient behaviors. Resilience theory suggests that stress may be moderated by various resources and processes, however, few studies have examined factors that may influence emotional resilience among Black family caregivers. Spearman’s rank correlation coefficient was used to assess the relationships between adaptive coping, caregiving self-efficacy, and positive affect within a sample of current caregivers (n=20). Self-efficacy in obtaining respite care was significantly correlated with both positive affect (p=.041) and adaptive coping (p=.022). Self-efficacy for managing difficult behaviors was also correlated with positive affect (p=.001). Finally, we identified a significant correlation between body mass index and self-efficacy for controlling upsetting thoughts. Caregiver resilience may be enhanced by providing increased access to respite services and culturally responsive support programs aimed at enhancing positive emotions and affective balance.

